# The Role of Non-genetic Therapies to Reduce the Incidence of Sickle Cell Crisis: A Systematic Review

**DOI:** 10.7759/cureus.42785

**Published:** 2023-08-01

**Authors:** Shravya Pingili, Vijaya Krishna Makkena, Arturo P Jaramillo, Babatope L Awosusi, Javaria Ayyub, Karan Nareshbhai Dabhi, Namra V Gohil, Nida Tanveer, Sally Hussein, Pousette Hamid

**Affiliations:** 1 Internal Medicine, California Institute of Behavioral Neurosciences & Psychology, Fairfield, USA; 2 Pathology and Laboratory Medicine, California Institute of Behavioral Neurosciences & Psychology, Fairfield, USA; 3 Neurology, California Institute of Behavioral Neurosciences & Psychology, Fairfield, USA

**Keywords:** anti sickling agents, blood transfusions, crizanlizumab, l- glutamate, phyto pharmacology, sickle cell disease (scd), vaso occlusive crisis, voxelotor, zinc

## Abstract

Sickle cell anemia is a hemoglobinopathy that causes complications such as Vaso-Occlusive Crisis (VOC), stroke, priapism, Acute Chest Syndromes (ACS), and bone infarcts due to blood vessel occlusion, resulting in hypoxia, ischemia, and inflammation. Preventing these incidents improves the quality of life and lowers mortality rates in Sickle Cell Disease (SCD) patients. This systematic review aims to describe the drugs, their mechanisms of action, dosages, changes in hemoglobin parameters, decrease in VOCs, delay the time for the next VOC, decrease in the length of hospital stay, and side effects associated with these drugs. This review adheres to the Preferred Reporting Items for Systematic Review and Meta-Analysis (PRISMA) 2020 guidelines. For this review, we searched the PubMed, Google Scholar, and Cochrane databases and screened them for full free texts published in English and studied in humans in the last five years beginning in 2018. Randomized clinical trials (RCT), observational studies, meta-analyses, systemic reviews, and traditional reviews were all included in the search. According to the type of study, quality assessment tools are used, and eight papers are chosen. Full-text articles from these papers are studied, analyzed, and tabulated. We discussed seven interventions that are used to treat sickle cell disease. Voxelotor, crizanlizumab, L-glutamate, long-term blood transfusions, Zinc (Zn), Niprisan®, and Ciklavit* were found to reduce the number and severity of VOC. We discovered that VOCs containing L -glutamate reduced the length of hospitalization. Magnesium (Mg) did not affect the number and severity of VOCs. This review includes a few articles for the study. Future papers on this subject should include a large sample size and many papers. More clinical trials are required to evaluate the dosages and outcomes of using these drugs in combination to prevent VOCs.

## Introduction and background

Sickle cell anemia is caused by substituting glutamate for valine in the 600th codon of the hemoglobin gene [1]. The hemoglobins causing sickle cell anemia are Hb SS, Hb C, Hb SC, and Hb S beta-thalassemia. The substitution of lysine for glutamate results in Hb C [2]. Sickle cell anemia affects approximately one in 500 African Americans and one in 36,000 Hispanic Americans [3]. The problem with sickle cell anemia is that the hemoglobin polymerizes and sickles under conditions such as hypoxia, infection, dehydration, and cold temperatures [4]. This mechanism results in a VOC, whose complications are Acute Chest Syndromes (ACS), stroke, priapism, and splenic sequestration.

Blood transfusions are a common treatment for Sickle Cell Disease (SCD) in developing countries. However, they have recently begun using hydroxyurea to prevent sickling. Despite using both, there is no reduction in VOC and other SCD complications. Numerous clinical trials have used a combination of drugs to prevent these episodes. Voxelotor, crizanlizumab, and L-glutamate were recently approved by the Food and Drug Administration (FDA). Other agents used are Magnesium (Mg) and Zinc (Zn).

Phytomedicines and gene replacement therapies using CRISPR (clustered regularly interspaced short palindromic repeats) Caspase techniques are currently being tested for SCD. Despite advances in SCD management, VOC remains the leading cause of death and morbidity [5].

## Review

Methodology

The systematic review follows the Preferred Reporting Items for Systematic Review and Meta-Analysis (PRISMA) 2020 guidelines [6]. This systematic review was conducted using PubMed, Google Scholar, and Cochrane with the keywords "Sickle cell anemia" and "Anti-sickling agents" in all three databases, as well as Medical Subject Headings (MeSH) terms and keywords in PubMed Search, and multiple filters for each journal. These are shown in Table 1 below.

**Table 1:** Databases used, keywords, search strategy and filters applied Hb: Hemoglobin, RCT: Randomized control trials

Databases	Keywords	Search strategy	Filters applied	Search results
PubMed	Sickle cell anemia or Sickle cell disease or Hemoglobin S or Hbs disease or Sickle cell trait AND Anti sickling agents	Sickle cell anemia OR Sickle cell disease OR Hemoglobin S OR Hbs disease OR Sickle cell trait OR ( "Anemia, Sickle Cell/blood"[Majr] OR "Anemia, Sickle Cell/diet therapy"[Majr] OR "Anemia, Sickle Cell/drug therapy"[Majr] OR "Anemia, Sickle Cell/prevention and control"[Majr] OR "Anemia, Sickle Cell/rehabilitation"[Majr] OR "Anemia, Sickle Cell/therapy"[Majr] ) AND Anti sickling agent OR ( "Antisickling Agents/administration and dosage"[Majr] OR "Antisickling Agents/blood"[Majr] OR "Antisickling Agents/pharmacology"[Majr] OR "Antisickling Agents/therapeutic use"[Majr] )	1. Free full text, 2. Last 5 years, 3. Humans, 4. English, 5. Article type, a) Randomized control trials (RCT), b) Observational studies, c) Meta-analysis, d) Systematic Review, e) Traditional review	1265 (last searched on April 12, 2023)
Google Scholar	Sickle cell anemia or Sickle cell disease or Hemoglobin S or Hbs disease or Sickle cell trait AND Anti sickling agents	Sickle cell anemia or Sickle cell disease or Hemoglobin S or Hbs disease or Sickle cell trait AND Anti sickling agents	Publications from 2018 to 2023, screened the first 200 articles	2480 (last searched on April 12, 2023)
Cochrane	Sickle cell anemia or Sickle cell disease or Hemoglobin S or Hbs disease or Sickle cell trait AND Anti sickling agents	[Anemia, Sickle Cell] explode all trees and with qualifier(s): [drug therapy - DT, therapy - TH]	1) Free articles, 2) from 2018 to 2023, 3) English articles, 4) Review articles	22 (last searched on April 12, 2023)

Inclusion Criteria

We gathered peer-reviewed free full-text English papers published in the last five years, beginning in 2018, and studied humans worldwide. These articles include Randomized Controlled Trials (RCTs), observational studies, meta-analyses, systematic reviews, and narrative reviews.

Exclusion Criteria

We excluded papers from grey literature, animal studies, and papers published five years before the paper was written.

Selection Strategy

The articles were chosen independently by the reviewers who used the exact keywords. The articles were initially screened using titles and abstracts, and the full-text articles were read. If there were conflicting results regarding the eligibility of the article, reviewers assessed the full-text article until the group reached a consensus.

Risk of Bias Assessment

The studies chosen for risk of bias assessment were reviewed independently by two reviewers using respective risk assessment tools, and only studies scoring more than 70% are included in this review. The tools used for each study under consideration are listed in Table 2 below.

**Table 2:** Risk of Bias Assessment CCBRT: Cochrane Collaboration Risk of Bias Tool, AMSTAR: Assessment of Multiple Systematic Reviews, SANRA: Scale for the Assessment of Narrative Review Articles

Quality assessment tool	Type of study	Total score	Accepted score (>70%)	Accepted studies
Cochrane Collaboration Risk of bias tool (CCRBT)	Randomized control Trial	7	5	Three studies. Hutchaleelaha et al., 2019 [7], Kutlar et al., 2019 [8], Niihara et al., 2018 [9]
Modified New Castle OTTOWA Scale	Clinical trials	5	6	0
Assessment of Multiple Systematic Reviews (AMSTAR) Checklist	Meta-analysis	11	8	Three Studies. Than et al., 2019 [10], Fortin et al., 2018 [11], Nagalla & Ballas, 2018 [12]
Scale for the Assessment of Narrative Review Articles (SANRA) Checklist	Traditional review	12	9	Two studies. Oniyangi & Cohall, 2020 [13], Ali et al., 2020 [14]

Results

**Figure 1:**
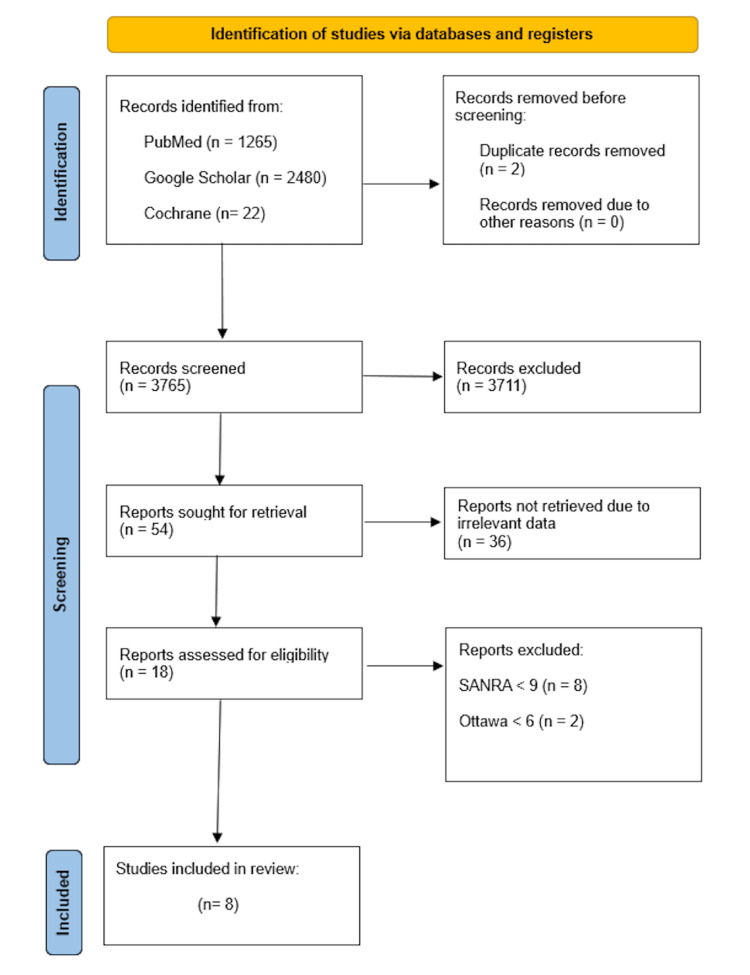
Flow Chart of the study selection process SANRA: Scale for the Assessment of Narrative Review Articles, Ottawa: Modified New Castle Ottawa Scale

There were a total of 3767 articles found according to the database search. Out of which, we got 3765 articles after removing duplicates. When the titles and extracts of these articles were evaluated based on the inclusion and exclusion criteria for this review, 54 articles were retrieved. From these, 36 were discarded due to irrelevant data. Eight papers with scores more than 70% were allowed to the publication after each publication underwent a quality assessment. There were three RCTs, three meta-analyses, and two traditional reviews. A flow diagram of the articles' selection and screening process is shown in Figure 1 below [15].

The main characteristic of the three RCTs, three meta-analyses, and two narrative reviews are shown in the table below. Of these eight studies, one RCT is about voxelotor, one about crizanlizumab, and the other RCT is about L-glutamate. One meta-analysis was focused on Magnesium, another was about blood transfusions, and another was about the usage of Zinc in preventing the sickle cell crisis. Of the two narrative reviews, one was focused on phytomedicines, while the other was about pharmacological interventions like voxelotor, crizanlizumab, and L-glutamate. The characteristics of these studies are shown in Table 3 below.

**Table 3:** Study characteristics of clinical trials, meta-analysis and narrative review RCT- Randomized Controlled Trial, VOC - Vaso Occlusive Crisis, RBC - Red Blood Cell, IV - Intra Venous, SCD - Sickle Cell Disease

Study	Type of Study	Population	Intervention	Duration of the study	Results	Follow-up of the Intervention
Hutchaleelaha et al., 2019 [7]	RCT	Sickle cell disease patient (HbSS, HbS/Beta Thalassemia, HbSC) with concomitant use of hydroxyurea with a stable dosage for greater than three months	Six Sickle cell patients received a single dose of Voxelotor 1000mg daily, and 2 received a placebo. Healthy volunteers were given Voxelotor a single ascending dose (SAD) in cohorts ranging from 100 mg to 2800 mg, and other groups received multiple increasing doses of Voxelotor up to 900 mg.	NA	1. Voxelotor once-daily oral dosing demonstrated increased affinity between oxygen and hemoglobin. 2. Voxelotor is well tolerated, with no dose-limiting toxicities or indication for hypoxia.	Safety and tolerability are monitored every 27 days following the completion of single doses and after 45 days following completion of the multiple-dose.
Kutlar et al., 2019 [8]	RCT	Sickle cell population aged 16- 65 years receiving hydroxyurea for > 6 months at a stable dose in the previous three months	Sickle cell patients were grouped into three cohorts receiving Crizanlizumab doses of 2. 5/kg or 5 mg/kg or placebo along with hydroxyurea	IV Crizanlizumab 14 times over 52 weeks. Patients were given two loading doses in the first month followed by monthly infusions.	1. Increased VOC-free intervals in patients taking 5mg/kg Crizanlizumab vs placebo. 2. Increased time for the first episode of VOC in patients taking Crizanlizumab.	There was a follow-up evaluation phase of six weeks.
Niihara et al., 2018 [9]	RCT	Sickle cell patients (Hb SS, Hb S/ Beta thalassemia) aged 5-58 with at least 2 pain crises who received hydroxy urea at a stable dose for at least three months.	Sickle cell patients were given L-glutamate or placebo powder along with hydroxyurea	Oral L-glutamate or placebo powder mixed in non-heat liquid. 0. 3g/kg powder was given twice daily for 48 weeks followed by a taper for three weeks	1. Decrease in pain crises over 48 weeks for those who receive L -glutamate regardless of hydroxyurea. 2. Based on this phase 3 trial, FDA approved pharmaceutical L- glutamine as a prescription to decrease VOC	Participants were contacted over the telephone weekly between monthly follow-ups at clinics.
Than et al., 2019 [10]	Meta-analysis	Two studies included adults and children, while three studies had paediatric patients. All of them were HbSS or HBSC or HbS/ beta Thalassemia.	1. In two studies Intravenous Magnesium sulphate was compared to normal saline for hospitalized patients for VOC. 2. Oral Magnesium Pidolate	IV Mg was given only during the stay in the hospital, while Oral Mg is given for six months	Decrease in the length of hospital stay and increase in quality of life in children	Patients who received IV Mg were assessed using daily pain scores during their hospital stay.
Fortin et al., 2018 [11]	Meta-analysis	All ages of Sickle cell anemia patients	1. RBC transfusions plus standard care versus standard care 2. RBC transfusions versus disease-modifying agents (hydroxyurea) 4. Liberal vs Restrictive blood transfusions 3. RBC transfusion vs no RBC transfusions	NA	The study showed a decreased risk of stroke in children and adolescents at high risk of stroke or silent cerebral infarcts.	6 months to 3 years.
Nagalla & Ballas, 2018 [12]	Meta-analysis	Sickle cell anemia patients aged 12 to 65 years who were on hydroxyurea with a stable dose in the last 3 months	They used Zinc sulphate in one study and senicapoc was used in the other two studies.	1. Oral Zinc sulphate was used for 18 months 2. Oral senicapoc was used for 54 weeks.	1. Low-quality evidence shows that Zinc sulphate reduces pain crises in sickle cell patients. 2. Senicapoc improves red cell survival in a dose-dependent manner, but the laboratory outcomes did not translate into clinical outcomes. So, the drug cannot be used in clinical practice to prevent VOC.	1. The participants in the oral Zinc sulphate study were followed up weekly. 2. The participants in the Senicapoc study were followed up for 8 weeks after the intervention.
Oniyangi & Cohall, 2020 [13]	Narrative review	Sickle cell patients of any age who were on or not on any medications	1. Niprisan®/Nicosan®, 2. Ciklavit*, 3. extract from Pfaffia paniculata along with any other medication they were taking	1. Niprisan®: Single daily dose of 12mg/kg for six months and then switched to placebo for another six months 2. Ciklavit*: In <5 years, 10 ml twice daily for six months. In >5 years, 20 ml twice daily for six months. 3. Pfaffina paniculata: Two 500 mg powdered capsules eight hours apart for three months.	1. Niprisan®: It is safe and effective in decreasing VOC episodes. There are no major adverse effects. 2. Ciklavit®: It has little or no effect in reducing VOCs and has a possible adverse effect of anemia. 3. Pfaffia paniculata: There were no clear outcomes about the outcomes	1. Niprisan: The study population was followed weekly over the telephone and then monthly visits at the clinic for 6 months. 2. Ciklavit: The patients were followed monthly for 6 months. 3. Pfaffina Paniculata: Patients were followed after 3 months.
Ali et al., 2020 [14]	Narrative review	1. Voxelotor 2. L-glutamate 3. Crizanlizumab	Voxelotor, L-glutamate, Crizanlizumab	-	While gene therapy and hemopoietic stem cell transplantation (HSCT) are still under trial pharmacological management like L-glutamate and Crizanlizumab reduce the number of VOC episodes and hospitalizations, regardless of hydroxyurea use. However, these two drugs do not improve haemoglobin levels. On the other hand, voxelotor improves hemoglobin levels and prevents hemolysis in SCD patients regardless of hydroxyurea use.	-

Outcomes

The number of VOC episodes, the time between these episodes, the improvement in oxygen hemoglobin affinity, and the left shift of the oxy-hemoglobin dissociation curve are used to assess the effectiveness of the new interventions. Each article used one or more of these interventions to describe the outcomes.

Discussion

Red Blood Cell (RBC) predisposes to hemoglobin precipitation on the membrane, which causes the release of iron-dependent free radicals and therefore the membrane damage finally resulting in the release of inflammatory markers [16]. This damaged membrane makes the RBC adhere more to the blood vessel endothelium, forming a nidus and obstructing blood flow.

The pathophysiology of VOC is not solely attributed to the occlusion of blood vessels; it can also be driven by intimal hyperplasia of large vessels, thrombus formation in blood vessels, and fat embolization of bone marrow. This may bring about hypoxia, reperfusion injury, ischemic tissue damage, and inflammation. The other mechanisms involved in VOC are adherence to circulating blood elements, such as leukocytes, to endothelial cells, hypercoagulability, endothelial dysfunction, altered nitric oxide (NO) metabolism, and ischemia-reperfusion injury [17].

VOCs can be triggered in a variety of ways. The patient factors include hypoxia, infection, fever, acidosis, dehydration, pregnancy, menstruation, obstructive sleep apnea, pain, anxiety, depression, alcohol consumption, and physical exhaustion. The environmental factors include exposure to temperature extremes, high wind speed, and humidity [17].

There are four phases of VOC [18,19]. Phase 1 is a prodromal period that precedes the actual VOC. It usually occurs 3-4 days prior to the actual pain. Fatigue, dizziness, weakness, swelling and tenderness of hands and feet, change in appetite, pallor, and jaundice are all symptoms. The real pain begins in phase 2, the evolving phase. Symptoms include waxing and waning pain in a single location. The hematological parameters revealed an increase in RBC density, a decrease in hemoglobin, and the amount of RBC deformation. The pain becomes more intense and peaks when entering phase 3 of VOC, necessitating hospitalization. The pain lasts 4-5 days. The patient describes it as stabbing and unbearable pain [18,19]. The hematological parameters revealed increased reticulocytes, inflammatory markers, RBC density, and Hb.

The final phase, phase 4, is a four-day recovery or post-crisis phase. The pain is relieved initially by intravenous fluids and then gradually over time. The hemoglobin parameters are restored. The improvement in RBC deformability and a decrease in dense cells at the end of a crisis are new risk factors that may cause a recurrence of VOC within a month in approximately 50% of painful episodes [18,19].

VOC prevention entails focusing on the triggering events and the various stages of the vicious cycle that leads to the condition. Many clinical trials were deemed ineffective. Until 2019, we only have hydroxyurea to prevent VOCs. In the following paragraphs, we will discuss voxelotor, crizanlizumab, L-glutamate, Magnesium, Zinc, phytotoxins, and long-term vs prophylactic blood transfusions to prevent RBC sickling.

Voxelotor

It is a disease-modifying agent that increases the affinity of oxygen to hemoglobin [20]. Oxygenated Hb is a potent inhibitor of RBC sickling. Voxelotor is also known as GBT440, a novel molecule for hemoglobin modification [7]. In our review, two papers were included regarding voxelotor. One is a phase 3 clinical trial, and the other is a narrative review. The phase 3 human clinical trial assesses the voxelotor’ s safety, efficacy, pharmacokinetics, and pharmacodynamics. The trial aims to see if single dosing aids in achieving the primary outcomes. Single and multiple doses of voxelotor to healthy patients and a single dose to SCD patients were given. The clinical trial found that once-daily oral dosing of Voxelotor had a linear dose-response relationship with the drug's pharmacokinetics [7].

The pharmacokinetic analysis looked at the levels of voxelotor in the blood, RBCs, and plasma. The Hb-oxygen affinity was used to measure pharmacodynamics. A reasonable oral dose of 900 mg of voxelotor achieves a target Hb modification of 40% in healthy volunteers. The maximum single-time oral dose given to an SCD patient is 1000mg, and the healthy volunteer is 2800mg, both of which are well tolerated in these patients. The drug had a long half-life, which allowed for single dosing. No dose-related toxicities were reported due to the drug's high RBC to plasma partitioning. The reported side effects are abdominal pain, diarrhoea, gastroenteritis, dizziness, and headache. The voxelotor is administered orally at 1500mg once daily to patients over the age of 12 years [20].

Crizanlizumab

It is a monoclonal antibody that prevents p-selectin on the endothelium and platelets from interacting with RBCs and leukocytes [21]. In our review two papers were included regarding crizanlizumab, one is a clinical trial and the other is a narrative review. The purpose of this study is to see if crizanlizumab has any meaningful clinical outcomes in terms of the number of VOC, increased time for the next VOC, and efficacy of low dose vs high dose. The study lasted 52 weeks, with serum concentrations of crizanlizumab ranging from 2.8 to 6.8 g/mL with a low dose to 10.5 to 15 g/mL with a high dose. The median annual VOC rate in those receiving concomitant hydroxyurea is 2.43 vs 3.58 in the placebo group, representing a 32.1% lower rate. However, the median times to first and second VOC were found to be statistically significantly longer in those receiving high-dose crizanlizumab versus those receiving placebo. The median time to VOC in the low-dose crizanlizumab group did not differ significantly from the placebo. Compared to the placebo, the high-dose crizanlizumab group showed 62.9% fewer uncomplicated crises per year [8]. Some of the serious adverse effects in the group included pyrexia, influenza, and pneumonia. It is approved for use in patients aged 16 years and up and is administered via intravenous infusion over 30 minutes, initially every two weeks and then every four weeks [8,21].

L-Glutamate

In our review two papers were included regarding L-glutamate, one is a phase 3 clinical trial, and the other is a narrative review. The study aims to count the number of VOCs, the number of hospitalizations, and the changes in hematological parameters in patients taking L-glutamate with or without hydroxyurea. Patients were given 0.3mg/kg glutamate for 48 weeks in this study, and they found a significant difference in median VOC in the treatment and control group (3 vs 2). The difference in median hospitalizations between treatment and control groups is statistically significant (2 vs 3, p=0. 05). The difference in hospitalization duration between the treatment and control groups is statistically significant (11 vs 7, p=0. 05). The drug has the advantage of not requiring any monitoring for adverse events. The disadvantages of L-glutamate are that it is expensive and must be mixed with any other beverage. There are issues with combining L-glutamate and hydroxyurea. Based on the results of this study, the FDA-approved pharmaceutical L-glutamate under the brand name Endari (Emmaus Medical) for the treatment of VOC. It can be given to patients over the age of five [9].

Magnesium

Intravenous (IV) Magnesium acts in two ways. One of them is calcium inhibition on the smooth muscles of blood vessels, and the other is the release of Nitic oxide from the endothelium [22]. Oral Mg assists in ion exchange at the cell membrane, such as the Sodium-Magnesium (Na-Mg) exchange, Potassium-Chloride (K-Cl) cotransport, and Sodium-Potassium (Na-K) ATPase.

This review article includes a meta-analysis describing various studies on IV Mg and oral Mg. The study aims to determine the efficacy of IV Mg in reducing the length of hospitalization and the quality of outcome, as well as the efficacy of oral Mg in reducing the number of VOCs and the quality of life in children and adults. When compared to the placebo, neither the IV Mg nor the oral Mg demonstrated a significant difference in improvement. The study is of moderate to low-quality evidence [10].

We can expect dose-dependent adverse events with Magnesium. Patients taking Mg get nausea, flushing, headache, lethargy, drowsiness, and decreased deep tendon reflexes at 4-6meq/l. They get somnolence, hypocalcaemia, absent deep tendon reflexes, hypotension, bradycardia, and electrocardiogram (ECG) changes at 6-10meq/l. At >10meq/l, patients experience muscle paralysis, apnea and respiratory failure, complete heart block, and cardiac arrest [23]. So, we should be cautious while administering magnesium.

Blood Transfusions

This review paper includes one meta-analysis that discusses the reviews on blood transfusion strategies, short-term/long-term, and restrictive versus liberal transfusions. It also compares the outcomes with standard care, treatments for complications, and disease-modifying agents such as hydroxyurea. The outcomes sought are serious adverse events from sickle cell complications such as stroke, ACS, Silent Cerebral Ischemia (SCI), and pain crises.

In children with abnormal Trans Cranial Dopplers (TCD), Long-term transfusions versus standard care may reduce SCI and ACS but do not affect pain crises. We have no data on whether chronic blood transfusions or disease-modifying therapy improves these parameters. There is no change in SCIs with hydroxyurea and phlebotomy. We do not know if prophylactic RBC transfusions lower the risk of ACS but may lower the likelihood of a painful crisis [11]. Further research and reporting on the effect of blood transfusion in preventing complications and improving the quality of life in adults is required [17].

Preventing Dehydration of RBC

This review paper included one study comparing the effectiveness of Zinc against a placebo. The study compared the effectiveness of senicapoc to placebo and discussed other drugs that work on cell membranes to prevent RBC dehydration. Zinc antagonizes intracellular calcium, preventing RBC dehydration and improving RBC membrane status [24]. The modulation of transmembrane Na-K pumps is another mechanism of Zinc. Dipyridamole is one of the medications that work via Na-K pumps to keep RBCs hydrated. Iron deposits damage the cell membrane and open Na-K pumps, resulting in dehydration [25]. In these situations, iron chelators come in handy. Arginine, an amino acid, releases NO, which reduces RBC density via the Gordon channel.

The review aims at studying the mortality rate, the reduction of VOC, and other SCD-related complications. Senicapoc improved RBC survival but did not affect clinical outcomes [12]. There was no significant difference in the reduction of VOCs in the senicapoc treatment group vs placebo (0.38 vs 0.31, p = 0. 054). The senicapoc phase 3 trial was cancelled because the results of the phase 2 trial did not meet the primary endpoint [26].

In the Zinc treatment group, there is a significant difference in painful crisis (1.40 vs 3.38) [12]. To supplement the evidence further research is needed on Zinc's mechanism of action in treating SCD.

Phytomedicines

This review paper included one study which discusses the risks and benefits of phytomedicines such as Niprisan®/Nicosan®, Ciklavit*, and powdered extract of Pfaff paniculata. In hypoxic conditions, Niprisan® retards Hb polymerization and reverses sickling [27]. Pfaff paniculata is a Sodium Ionophore that improves hydration and deformation in the RBC.

The primary outcomes studied were the frequency and severity of pain crises, the frequency of severe complications, and the frequency of hospital admissions due to complications. In a phase 2 trial of Niprisan®, there was a significant difference in the frequency and severity of pain crises (7.9 vs 21.1). Ciklavit* has shown a reduction in the frequency and severity of pain crises compared to placebo (4.4 vs. 4.2). Ciklavit* caused anemia in some patients. Pfaff paniculata improved in several parameters such as Mean Cell Volume (MCV), Mean Cell Hemoglobin (MCH), reticulocytes, and foetal hemoglobin, and even the number of sickle cells decreased. However, no comparisons are made between Pfaff paniculata and the placebo group [13]. More research into phytomedicines is required for concrete evidence to prescribe dosages.

## Conclusions

Current knowledge of VOC prevention is limited to the application of hydroxyurea. The recently FDA-approved drugs voxelotor, crizanlizumab, and L-glutamate, as well as other treatments such as Zinc, Magnesium, phytomedicines, and blood transfusions, are effective in the prevention of VOCs by addressing changes in various parameters such as the hemoglobin, median length of stay during an episode, and the time interval between episodes, according to our review. This review's primary objective is to encourage readers to transfer their attention from hydroxyurea to these newer drugs and their combination to prevent VOCs. There is a need for additional research into the drug interactions, adverse effects, and dosages of these drugs when used in combination with each other or with hydroxyurea. This systematic review has limitations; only a few studies with small sample sizes were included. Future study inclusion criteria must include a large number of studies and sample sizes. More randomized controlled trials (RCTs) on these pharmaceuticals are required to advance our knowledge.
