# Correction: The Role of Non-genetic Therapies to Reduce the Incidence of Sickle Cell Crisis: A Systematic Review

**DOI:** 10.7759/cureus.c302

**Published:** 2025-09-23

**Authors:** Shravya Pingili, Vijaya Krishna Makkena, Arturo P Jaramillo, Babatope L Awosusi, Javaria Ayyub, Karan Nareshbhai Dabhi, Namra V Gohil, Nida Tanveer, Sally Hussein, Pousette Hamid

**Affiliations:** 1 Internal Medicine, California Institute of Behavioral Neurosciences & Psychology, Fairfield, USA; 2 Pathology and Laboratory Medicine, California Institute of Behavioral Neurosciences & Psychology, Fairfield, USA; 3 Neurology, California Institute of Behavioral Neurosciences & Psychology, Fairfield, USA

This article has been corrected to fix a minor typo in the PRISMA diagram: The number of records excluded was originally incorrectly listed as 3,712. This has been corrected to 3,711.

